# A Comparison of Estimation Methods for a Multi-unidimensional Graded Response IRT Model

**DOI:** 10.3389/fpsyg.2016.00880

**Published:** 2016-06-10

**Authors:** Tzu-Chun Kuo, Yanyan Sheng

**Affiliations:** Department of Counseling, Quantitative Methods, and Special Education, Southern Illinois University CarbondaleCarbondale, IL, USA

**Keywords:** item response theory, multi-unidimensional model, Markov chain Monte Carlo, MML, fully Bayesian, graded response model, IRTPRO, BMIRT

## Abstract

This study compared several parameter estimation methods for multi-unidimensional graded response models using their corresponding statistical software programs and packages. Specifically, we compared two marginal maximum likelihood (MML) approaches (Bock-Aitkin expectation-maximum algorithm, adaptive quadrature approach), four fully Bayesian algorithms (Gibbs sampling, Metropolis-Hastings, Hastings-within-Gibbs, blocked Metropolis), and the Metropolis-Hastings Robbins-Monro (MHRM) algorithm via the use of IRTPRO, BMIRT, and MATLAB. Simulation results suggested that, when the intertrait correlation was low, these estimation methods provided similar results. However, if the dimensions were moderately or highly correlated, Hastings-within-Gibbs had an overall better parameter recovery of item discrimination and intertrait correlation parameters. The performances of these estimation methods with different sample sizes and test lengths are also discussed.

## 1. Introduction

Polytomous item response theory (IRT; Lord, 1980) models are applicable for tests with items involving more than two response categories. Polytomous responses include nominal and ordinal responses. The former does not have any natural ordering between categories whereas the latter corresponds to a number of ordering categories. Ordinal polytomous responses, such as Likert scale items (Likert, 1932), are broadly used in many fields, including education, psychology, and marketing. Given this, many IRT models have been developed to analyze ordinal polytomous items, such as the graded response model (GRM; Samejima, 1969), the rating scale model (RSM; Andrich, 1978), and the partial credit model (PCM; Masters, 1982), to name a few. This study focuses on the GRM, the most widely used IRT model for polytomous response data (e.g., Rubio et al., 2007; Ferero and Maydeu-Olivares, 2009). The unidimensional GRM is defined as

(1) $$\begin{array}{l} P\left(Y_{ij}=c|\theta_{i},\delta_{j}\right)=P\left(Y_{ij}\geq c-1|\theta_{i},\delta_{j}\right)-P\left(Y_{ij}\geq c|\theta_{i},\delta_{j}\right)\;\\ =F\left(\alpha_{j}\theta_{i}-\delta_{j,c-1}\right)-F\left(\alpha_{j}\theta_{i}-\delta_{j,c}\right)\;\\ =\int_{\delta_{j,c-1}}^{\delta_{j,c}}f\left(z;\alpha_{j}\theta_{i}\right)dz,\; \end{array}$$

*i* = 1, …, *N, j* = 1, …, *K, c* = 1, 2, …, *C*_*j*_, where *F* and *f* denote the logistic or normal CDF and PDF for logistic or normal ogive GRM, θ_*i*_ denotes the person trait parameter, α_*j*_ denotes the item discrimination parameter, and δ_*j, c*_ denotes the item threshold parameter for the *c*th category response of item *j* (Samejima, 1969), the latter of which satisfies

(2) $$\begin{array}{l} -\infty =\delta_{j,0}<\delta_{j,1}<\ldots <\delta_{j,C_{j}-1}<\delta_{j,C_{j}}=\infty .\; \end{array}$$

In many circumstances, it suffices to assume that all the test items measure one trait in common and hence use unidimensional IRT models. However, in some situations when multiple traits are being measured or the test dimensionality structure is not clear, multidimensional IRT (MIRT; Reckase, 1997, 2009) models have to be considered. MIRT models are adopted when distinct multiple traits are involved in producing the manifest responses for an item. A special case of the MIRT model applies to the situation where the instrument consists of several subscales with each measuring one latent trait, such as the Minnesota Multiphasic Personality Inventory (MMPI; Buchanan, 1994). In the IRT literature, such a model is called the *multi-unidimensional* (Sheng and Wikle, 2007) or the *simple structure* MIRT (McDonald, 1999) model and is the major focus of the study.

The multi-unidimensional GRM applies to situations where a *K*-item instrument consists of *m* subscales or dimensions, each containing *k*_*v*_ polytomous response items that measure one latent dimension. The model is defined as:

(3) $$\begin{array}{l} P\left(Y_{vij}=c|\theta_{vi},\alpha_{vj},\delta_{j}\right)=F\left(\alpha_{vj}\theta_{vi}-\delta_{j,c-1}\right)-F\left(\alpha_{vj}\theta_{vi}-\delta_{j,c}\right)\;\\ =\int_{\delta_{j,c-1}}^{\delta_{j,c}}f\left(z;\alpha_{vj}\theta_{vi}\right)dz,\; \end{array}$$

*i* = 1, …, *N, j* = 1, …, *K, c* = 1, 2, …, *C*_*j*_, where *F* and *f* denote the logistic or normal CDF and PDF for logistic or normal ogive GRM, α_*vj*_ and θ_*vi*_ denote the item discrimination and the person's latent trait in the *v*th dimension, and δ_*j, c*_ denotes the item threshold parameter for the *c*th category response of item *j* (Samejima, 1969), the latter of which satisfies

(4) $$\begin{array}{l} -\infty =\delta_{j,0}<\delta_{j,1}<\ldots <\delta_{j,C_{j}-1}<\delta_{j,C_{j}}=\infty .\; \end{array}$$

For decades, IRT models can be estimated using (1) the marginal maximum likelihood (MML) estimation based on the expectation maximization (EM) algorithm and (2) the fully Bayesian estimation via the use of the Markov chain Monte Carlo (MCMC) simulation techniques. More recently, Cai (2010a,b) developed a Metropolis-Hastings Robbins-Monro (MHRM) algorithm, which combines the blocked Metropolis algorithm (Patz and Junker, 1999b) with Robins–Monro (Robbins and Monro, 1951) to facilitate the maximum likelihood estimation. In the literature, studies have been conducted to compare the performances of these methods in estimating unidimensional dichotomous (e.g., Baker, 1998), unidimensional polytomous (e.g., Cowels, 1996) and multidimensional dichotomous (e.g., Han and Paek, 2014) models. However, little has been done to investigate them in estimating multidimensional polytomous models.

In view of the above, this study focuses on comparing different estimation methods of the multi-unidimensional GRM. Specifically, the following estimation methods are compared: Bock and Aitkin's two-step MML approach (BAEM; Bock and Aitkin, 1981) and adaptive quadrature estimation (ADQ; Schilling and Bock, 2005) in MML, Gibbs sampling (Geman and Geman, 1984), Metropolis-Hastings (MH; Hastings, 1970; Metropolis and Ulam, 1949), Hastings-with-Gibbs (HwG; Cowels, 1996), and blocked Metropolis algorithm (BM; Patz and Junker, 1999a,b) in fully Bayesian estimation, and the MHRM algorithm. Each of these methods is briefly described in Section 2.

Several computer programs have been developed for estimating parameters in the multi-unidimensional GRM. For example, BAEM and MHRM are implemented in IRTPRO (Cai et al., 2011), and MH is implemented in BMIRT (Yao, 2003). This study then uses these software packages for the corresponding estimation method. The performance in parameter recovery of each method is evaluated and compared using Monte Carlo simulations. The results of this study can provide researchers/practitioners with a set of guidelines on the use of MML, fully Bayesian, and their hybrid, MHRM in estimating multi-unidimensional GRMs under different sample size, test length, and intertrait correlation conditions.

## 2. Estimation methods

This study focuses on three general categories of estimation methods of multi-unidimensional GRMs: (1) MML, (2) fully Bayesian estimation, and (3) MHRM. A brief description of the major techniques in each category is provided below.

### 2.1. Marginal maximum likelihood (MML)

The two-step MML was developed by Bock and Aitkin (1981). After obtaining the joint probability (likelihood) of the item response vector given the person parameters, MML treats persons as random effects and derives a marginal probability of observing the item response vector by integrating the person effect out of the joint likelihood in order to separate item parameters from person parameters. Hence, in MML, item parameters are estimated using the expectation-maximum (EM) algorithm, and person parameters can be subsequently obtained using the estimated item parameters.

Bock and Aitkin's original algorithm uses a fixed Gauss-Hermite quadrature, which is limited for models with a lower dimension (Baker and Kim, 2004). However, as the number of dimension increases, the number of quadrature points increases exponentially and must be accommodated for by decreasing the number of quadrature in each dimension. To overcome this problem, Schilling and Bock (2005) suggested the use of an adaptive quadrature for better accuracy when a smaller number of quadratures per dimension is used. This method can be used for models with a moderate number of dimensions. These two MML methods for multi-unidimensional GRMs are directly implemented in IRTPRO (Cai et al., 2011).

### 2.2. Fully bayesian estimation

For the past two decades, fully Bayesian estimation via the use of the Markov chain Monte Carlo (MCMC) has gained an increased interest due to improved computational efficiency. In Bayesian analysis, prior information and all available data are integrated into posterior distributions (using Bayes rule) on which one can base on their inferences. Specifically,

(5) $$\begin{array}{l} P\left(\xi |y\right)\propto P\left(y|\xi \right)P\left(\xi \right),\; \end{array}$$

where **ξ** and ***y*** are the collections of all parameters and observed data, respectively. Accurate approximations of the posterior distribution, *P*(**ξ**|***y***), via the use of MCMC simulation techniques have made fully Bayesian estimation available for complex models, such as MIRT models.

There are two types of fundamental mechanisms among the MCMC algorithm: Gibbs sampling (Geman and Geman, 1984) and Metropolis-Hastings (MH; Hastings, 1970; Metropolis and Ulam, 1949). Gibbs sampling is adopted in situations when the full conditional distribution of each parameter can be derived in closed form. If any of the full conditional distribution does not have an obtainable form, MH can be used via choosing a proposal or candidate distribution by the current value of the parameters. Then a proposal value is generated from the proposal distribution and accepted in the Markov chain with a certain amount of probability.

The two methods can be combined to form the blocked Metropolis (BM) procedure (Patz and Junker, 1999a,b), where each parameter is sampled from its full conditional distribution and an MH step is subsequently used to accept/reject it. Hastings-within-Gibbs (HwG) is another form of the hybrid between Gibbs sampling and MH. As far as GRMs are concerned, Albert and Chib (1993) proposed a Gibbs sampler for the unidimensional model. Their approach can be easily extended to the multi-unidimensional model. Cowels (1996) proposed a HwG procedure by using a MH step within the Gibbs sampler developed by Albert and Chib (1993) for sampling the threshold parameters to improve mixing and accelerate convergence. Kuo and Sheng (2015) extended Cowels' approach to the more general multi-unidimensional model, where a constrained multivariate normal prior was assumed for **θ**, **θ** ~ *N*_*m*_(***0, P***), with ***P*** being a correlation matrix to resolve the model location and scale indetermination. Moreover, the MH algorithm has been developed and implemented in BMIRT, and the BM procedure is implemented in IRTPRO for the multi-unidimensional model.

### 2.3. Metropolis-hastings robbins-monro (MHRM)

More recently, Cai (2010a,b) developed a MHRM algorithm to combine fully Bayesian estimation with Robins–Monro (Robbins and Monro, 1951) technique to facilitate the maximum likelihood. Specifically, studies have shown that MML would cause the estimation process for MIRT models to be computationally intensive and often intractable when a large number of dimensions are involved (Cai, 2010a,b; Chalmers, 2012). The MHRM algorithm was consequently developed to overcome the problem of MML and provide useful estimates for data with a large number of items, many dimensions, or missing data. The MHRM estimation for the multi-unidimensional GRM can also be implemented in IRTPRO.

## 3. Simulation study

### 3.1. Simulated data

To compare the aforementioned methods in estimating the multi-unidimensional GRM, a Monte Carlo simulation study was carried out where tests with two subscales were considered so that the first half measured one latent trait (θ_1_) and the second half measured the other (θ_2_). In the study, three factors were manipulated: sample size (*N*), test length (*K*), and intertrait correlation (ρ). The choice of *N*, *K*, and ρ was based on previous studies with similar models. For example, when investigating multidimensional GRMs, the simulation studies in Fu et al. (2010) adopted *N* = 500, 1000; *K* = 10, 20, 30, ρ = 0.1, 0.3, …, 0.9 for dichotomous items and *N* = 1000; *K* = 20, ρ = 0.2, 0.4, …0.8, for polytomous items involving three categories. Working with dichotomous multi-unidimensional models, Sheng and Wikle (2008) adopted *N* = 1000, *K* = 18, ρ = 0.2, 0.5, 0.8 in the simulation studies, while Sheng and Headrick (2012) adopted *N* = 1000, *K* = 10, ρ = 0.2, 0.4, 0.6. Wollack et al. (2002) conducted simulation studies with nominal response models and they observed that parameter recovery was improved by increasing the test length from 10 to 30 items but that increasing the test length from 20 to 30 items did not produce a noticeable difference. Consequently, with our study, *N* polytomous responses (*N* = 500, 1000) to *K* items (*K* = 20, 40) were generated according to the multi-unidimensional GRM, where the population correlation between the two latent traits (ρ) was set to be 0.2, 0.5, or 0.8. Each item was set to be measured on three-scale Likert scales so that two threshold parameters were estimated for each item. The item discrimination parameters **α**_*v*_ were generated randomly from uniform distributions so that α_*vj*_ ~ *U*(0, 2). The threshold parameters δ_*j*1_ and δ_*j*2_ were sorted values based on those randomly generated from a standard normal distribution, i.e., δ_*j*1_ = min(*X*_1_, *X*_2_) and δ_*j*2_ = max(*X*_1_, *X*_2_), where *X*_1_, *X*_2_ ~ *N*(0, 1).

Each set of simulated data was analyzed using: (1) BAEM, (2) ADQ, (3) Gibbs, (4) MH, (5) HwG, (6) BM, and (7) MHRM. It is noted that methods (1) and (2) are MML estimations, (3)–(6) are fully Bayesian estimations, and (7) is a hybrid of MML and fully Bayesian estimation. Moreover, non-informative prior distributions were considered for **α**_*v*_ with fully Bayesian methods, and the variance of proposal distributions was adjusted to have an adequate acceptance rate for (4) and (5).

For the use of software, (3) and (5) were programmed in MATLAB^1^, (1), (2), (6), and (7) were executed using IRTPRO^2^, and (4) was implemented in BMIRT^3^. However, in BMIRT, the population intertrait correlation, ρ, needs to be specified to implement the model, which may not be applicable in real situations. Yao and colleagues recommended Akaikes information criterion (AIC; Akaike, 1987) as a criterion in determining ρ when the latent structure is not clear (Yao and Schwarz, 2006). They further suggested that ρ can be approximated by finding the observed correlations between sum scores of subtests (Yao and Boughton, 2007). In this study, we considered all these scenarios when implementing MH via BMIRT, and they are: (1) directly using the true ρ (TRUE), (2) via the use of AIC (AIC), and (3) approximating ρ using the correlation between two subscores (COR). Hence, altogether this study compared nine estimation methods/approaches under two sample size, two test length, and three intertrait correlation conditions.

Ten replications were carried out for each scenario, where root-mean-squared differences (RMSDs) and bias were used to evaluate the recovery of each item parameter. The 10% trimmed means of these measures were calculated across items to provide summary statistics.

## 4. Results

Tables 1–4 display the RMSD's and biases for estimating the model under the 12 test situations using the nine estimation methods. Inspection of these tables gives rise to the following results:

**Table 1:** **Average RMSD (white rows) and bias (gray rows) for estimating α, δ, and ρ when *N* = 500, *K* = 20**.

	**MATLAB**		**BMIRT**			**IRTPRO**			
	**Gibbs**	**HwG**	**TRUE**	**AIC**	**COR**	**BAEM**	**ADQ**	**MHRM**	**BM**
**True ρ = 0.2**									
α_1_	0.154	0.147	0.137	0.140	0.140	0.150	0.150	0.151	0.153
	0.065	0.057	0.040	0.041	0.038	0.048	0.048	0.054	0.060
α_2_	0.156	0.130	0.123	0.126	0.123	0.130	0.130	0.129	0.134
	0.048	0.039	0.025	0.027	0.026	0.036	0.037	0.040	0.043
δ_1_	0.1404	0.1326	0.115	0.536	0.112	0.135	0.135	0.134	0.136
	0.024	−0.052	−0.022	0.006	−0.022	−0.039	−0.038	−0.037	−0.046
δ_2_	0.134	0.110	0.101	0.602	0.097	0.108	0.108	0.108	0.107
	0.050	−0.023	−0.007	0.064	−0.006	0.001	0.001	0.003	−0.004
ρ	0.098	0.076	0.060	0.066	0.069	0.078	0.078	0.077	0.077
	0.058	0.012	0.010	−0.025	0.004	0.014	0.014	0.014	0.017
**True ρ = 0.5**									
α_1_	0.020	0.019	0.014	0.016	0.013	0.015	0.015	0.015	0.018
	0.060	0.046	0.008	0.003	−0.002	0.032	0.032	0.036	0.042
α_2_	0.019	0.020	0.016	0.021	0.018	0.024	0.024	0.025	0.026
	0.047	0.033	0.013	0.019	0.009	0.032	0.033	0.039	0.041
δ_1_	0.045	0.021	0.011	0.012	0.012	0.016	0.016	0.016	0.017
	0.139	−0.027	0.020	0.025	0.028	0.000	−0.000	0.009	0.009
δ_2_	0.052	0.020	0.009	0.011	0.012	0.017	0.017	0.019	0.018
	0.163	−0.009	0.038	0.046	0.049	0.046	0.046	0.056	0.051
ρ	0.001	0.001	0.008	0.022	0.004	0.001	0.001	0.001	0.001
	0.020	−0.023	−0.064	−0.145	−0.059	−0.019	−0.019	−0.015	−0.0139
**True ρ = 0.8**									
α_1_	0.154	0.139	0.135	0.135	0.141	0.135	0.136	0.138	0.138
	0.055	0.035	0.010	−0.009	−0.033	0.025	0.025	0.030	0.032
α_2_	0.158	0.135	0.131	0.126	0.123	0.133	0.133	0.139	0.137
	0.070	0.052	0.032	0.011	−0.004	0.036	0.037	0.042	0.039
δ_1_	0.167	0.154	0.106	0.109	0.106	0.435	0.119	0.113	0.120
	0.106	−0.080	−0.020	−0.020	−0.014	0.080	−0.042	−0.036	−0.043
δ_2_	0.187	0.137	0.095	0.101	0.098	0.113	0.113	0.110	0.113
	0.137	−0.052	−0.003	−0.000	0.007	0.006	0.006	0.012	0.001
ρ	0.025	0.020	0.009	0.205	0.078	0.023	0.023	0.026	0.026
	0.019	0.000	0.003	−0.20	−0.072	0.008	0.008	0.013	0.012

**Table 2:** **Average RMSD (white rows) and bias (gray rows) for estimating α, δ, and ρ when *N* = 500, *K* = 40**.

	**MATLAB**		**BMIRT**			**IRTPRO**			
	**Gibbs**	**HwG**	**TRUE**	**AIC**	**COR**	**BAEM**	**ADQ**	**MHRM**	**BM**
**True ρ = 0.2**									
α_1_	0.216	0.148	0.120	0.121	0.115	0.132	0.131	0.132	0.125
	0.147	0.049	0.018	0.018	0.012	0.028	0.028	0.029	0.020
α_2_	0.227	0.184	0.121	0.120	0.118	0.129	0.129	0.130	0.129
	0.175	0.111	0.046	0.047	0.041	0.059	0.059	0.061	0.050
δ_1_	0.477	0.274	0.094	0.096	0.089	0.104	0.103	0.105	0.110
	0.410	−0.189	0.000	−0.005	−0.003	−0.018	−0.016	−0.024	−0.020
δ_2_	0.499	0.262	0.092	0.095	0.088	0.094	0.095	0.095	0.099
	0.429	−0.179	0.015	0.011	0.012	0.013	0.015	0.007	0.007
ρ	0.169	0.048	0.040	0.045	0.046	0.047	0.047	0.050	0.048
	0.163	0.018	0.001	−0.024	−0.003	0.001	0.001	0.001	0.001
**True ρ = 0.5**									
α_1_	0.180	0.102	0.140	0.143	0.140	0.108	0.107	0.112	0.108
	0.125	0.042	−0.071	−0.081	−0.0806	0.031	0.030	0.038	0.030
α_2_	0.180	0.111	0.096	0.099	0.097	0.117	0.116	0.118	0.121
	0.122	0.045	0.015	0.008	0.003	0.030	0.030	0.032	0.034
δ_1_	0.393	0.088	0.466	0.445	0.449	0.107	0.107	0.111	0.115
	0.324	−0.026	0.318	0.338	0.323	−0.018	−0.020	−0.044	−0.040
δ_2_	0.422	0.096	0.474	0.459	0.463	0.117	0.113	0.113	0.118
	0.344	−0.010	0.321	0.342	0.324	0.030	0.019	0.006	0.001
ρ	0.097	0.039	0.031	0.084	0.037	0.039	0.040	0.040	0.028
	0.092	−0.005	0.006	−0.073	−0.012	0.000	0.000	0.003	−0.009
**True ρ = 0.8**									
α_1_	0.243	0.097	0.126	0.139	0.150	0.096	0.096	0.099	0.096
	0.184	0.042	−0.072	−0.083	−0.104	0.025	0.025	0.028	0.017
α_2_	0.265	0.118	0.111	0.118	0.125	0.115	0.116	0.121	0.113
	0.187	0.036	−0.027	−0.011	−0.062	0.017	0.017	0.018	0.014
δ_1_	0.579	0.106	0.507	0.470	0.489	0.106	0.103	0.010	0.102
	0.495	−0.042	0.344	0.364	0.329	−0.027	−0.024	−0.004	−0.023
δ_2_	0.605	0.116	0.502	0.473	0.486	0.118	0.116	0.126	0.116
	0.511	−0.033	0.326	0.348	0.312	0.006	0.009	0.028	0.005
ρ	0.061	0.016	0.017	0.116	0.021	0.019	0.020	0.020	0.018
	0.058	0.004	0.013	−0.114	−0.015	0.011	0.011	0.012	0.012

**Table 3:** **Average RMSD (white rows) and bias (gray rows) for estimating α, δ, and ρ when *N* = 1000, *K* = 20**.

	**MATLAB**		**BMIRT**			**IRTPRO**			
	**Gibbs**	**HwG**	**TRUE**	**AIC**	**COR**	**BAEM**	**ADQ**	**MHRM**	**BM**
**True ρ = 0.8**									
α_1_	0.091	0.094	0.091	0.090	0.090	0.097	0.097	0.096	0.096
	0.016	0.016	0.018	0.018	0.019	0.026	0.027	0.030	0.023
α_2_	0.077	0.082	0.079	0.079	0.078	0.087	0.087	0.087	0.088
	0.023	0.020	0.024	0.021	0.025	0.030	0.030	0.029	0.028
δ_1_	0.097	0.099	0.094	0.099	0.098	0.108	0.109	0.104	0.111
	0.012	−0.051	−0.042	−0.045	−0.044	−0.061	−0.061	−0.052	−0.065
δ_2_	0.081	0.080	0.071	0.070	0.070	0.067	0.067	0.066	0.069
	0.013	−0.054	−0.029	−0.032	−0.031	−0.029	−0.029	−0.020	−0.036
ρ	0.036	0.028	0.020	0.041	0.022	0.029	0.029	0.029	0.029
	0.026	0.014	0.009	−0.031	0.003	0.013	0.013	0.013	0.012
**True ρ = 0.5**									
α_1_	0.194	0.080	0.087	0.086	0.080	0.097	0.097	0.098	0.097
	0.134	0.035	0.039	0.034	0.019	0.046	0.046	0.045	0.044
α_2_	0.146	0.073	0.069	0.073	0.136	0.076	0.076	0.076	0.078
	0.110	0.024	0.027	0.025	−0.050	0.031	0.031	0.034	0.029
δ_1_	0.435	0.082	0.098	0.098	0.095	0.108	0.107	0.102	0.105
	0.334	−0.024	−0.012	−0.019	−0.017	−0.030	−0.030	−0.018	−0.033
δ_2_	0.444	0.070	0.071	0.069	0.067	0.081	0.080	.078	0.078
	0.349	−0.018	0.009	0.004	0.005	0.008	0.008	0.019	0.003
ρ	0.102	0.036	0.022	0.142	0.058	0.035	0.036	0.038	0.035
	0.083	−0.000	0.002	−0.138	−0.037	0.003	0.003	0.003	0.003
**True ρ = 0.8**									
α_1_	0.138	0.078	0.082	0.079	0.074	0.089	0.089	0.090	0.087
	0.081	0.023	0.024	−0.014	−0.016	0.027	0.028	0.026	0.022
α_2_	0.156	0.094	0.089	0.086	0.082	0.097	0.097	0.192	0.096
	0.091	0.038	0.033	−0.010	−0.011	0.043	0.043	−0.041	0.039
δ_1_	0.334	0.078	0.082	0.161	0.079	0.074	0.074	0.074	0.078
	0.252	−0.039	−0.030	0.024	−0.023	−0.027	−0.027	−0.015	−0.023
δ_2_	0.355	0.075	0.066	0.203	0.065	0.071	0.071	0.074	0.074
	0.274	−0.025	−0.011	−0.059	0.000	0.002	0.003	0.015	0.004
ρ	0.030	0.008	0.004	0.201	0.065	0.011	0.011	0.011	0.011
	0.027	0.002	0.003	−0.193	−0.065	0.007	0.007	0.009	0.006

**Table 4:** **Average RMSD (white rows) and bias (gray rows) for estimating α, δ, and ρ when *N* = 1000, *K* = 40**.

	**MATLAB**		**BMIRT**			**IRTPRO**			
	**Gibbs**	**HwG**	**TRUE**	**AIC**	**COR**	**BAEM**	**ADQ**	**MHRM**	**BM**
**True ρ = 0.2**									
α_1_	0.405	0.084	0.122	0.126	0.124	0.094	0.095	0.091	0.094
	0.337	0.050	−0.052	−0.060	−0.058	0.054	0.054	0.049	0.053
α_2_	0.371	0.093	0.118	0.115	0.115	0.099	0.099	0.099	0.101
	0.308	0.046	0.026	0.028	0.023	0.050	0.050	0.049	0.050
δ_1_	0.867	0.106	0.504	0.501	0.501	0.081	0.081	0.083	0.080
	0.753	−0.024	0.385	0.395	0.382	−0.004	−0.004	0.009	0.001
δ_2_	0.899	0.122	0.512	0.511	0.506	0.107	0.107	0.114	0.108
	0.782	−0.007	0.365	0.376	0.363	0.040	0.040	0.052	0.043
ρ	0.300	0.049	0.046	0.052	0.052	0.048	0.048	0.046	0.047
	0.295	0.011	0.013	−0.016	0.012	0.010	0.011	0.011	0.010
**True ρ = 0.5**									
α_1_	0.375	0.073	0.104	0.106	0.109	0.083	0.083	0.088	0.086
	0.310	0.017	−0.052	−0.060	−0.065	0.023	0.023	0.023	0.028
α_2_	0.379	0.084	0.083	0.086	0.083	0.093	0.093	0.093	0.095
	0.330	0.026	0.013	0.009	0.006	0.031	0.031	0.030	0.036
δ_1_	0.892	0.084	0.537	0.524	0.533	0.091	0.090	0.093	0.078
	0.790	−0.020	0.379	0.410	0.382	−0.015	−0.014	−0.004	−0.005
δ_2_	0.928	0.083	0.533	0.531	0.530	0.092	0.092	0.101	0.092
	0.818	−0.003	0.402	0.433	0.405	0.031	0.032	0.042	0.032
ρ	0.196	0.026	0.022	0.071	0.026	0.027	0.027	0.028	0.028
	0.195	−0.001	0.004	−0.065	−0.009	0.002	0.002	0.003	0.004
**True ρ = 0.8**									
α_1_	0.413	0.082	0.079	0.092	0.095	0.089	0.089	0.089	0.094
	0.342	0.024	−0.019	−0.031	−0.046	0.027	0.027	0.025	0.032
α_2_	0.495	0.078	0.070	0.080	0.0690	0.086	0.087	0.088	0.095
	0.407	0.039	0.019	0.028	−0.011	0.039	0.039	0.040	0.046
δ_1_	0.955	0.105	0.475	0.454	0.459	0.080	0.079	0.081	0.081
	0.822	−0.036	0.338	0.357	0.332	−0.014	−0.012	0.007	−0.014
δ_2_	0.981	0.093	0.473	0.461	0.460	0.074	0.074	0.087	0.075
	0.841	−0.026	0.344	0.365	0.340	0.024	0.025	0.045	0.022
ρ	0.096	0.015	0.011	0.136	0.032	0.016	0.016	0.016	0.018
	0.095	0.001	0.007	−0.135	−0.023	0.005	0.005	0.004	0.007

The four methods implemented in IRTPRO (BAEM, ADQ, BM, and MHRM) yielded similar parameter estimates. Gibbs sampling had in general larger averaged RMSDs in recovering model parameters. This may be due to the slower convergence problem of Gibbs sampling as noted by Cowels (1996). It is noted that MH with each of the three criteria implemented in BMIRT (TRUE, AIC, COR) had a similar precision in estimating item discrimination (**α**) and threshold (**δ**) parameters. However, it only recovered ρ well under the TRUE criterion (where the population correlation was assumed to be available), which in practice is not realistic. On the other hand, MH with the COR criterion in BMIRT resulted in a slightly larger RMSD than the TRUE criterion. The AIC criterion had a worse performance on estimating ρ when the true intertrait correlation became larger.

These nine procedures performed differently in estimating **α** and **δ** under the 12 test situations. Specifically, when *N* = 500 and *K* = 20, the results shown in Table 1 indicated that MH with TRUE, AIC, or COR, as implemented in BMIRT had an overall better estimation of **α** and **δ** than the other procedures. HwG and the four procedures implemented in IRTPRO had similar results. Even though they did not estimate **α** and **δ** as accurately as MH, their RMSDs were not much larger.

When the test length was longer but the sample size remained the same (i.e., *N* = 500, *K* = 40). MH with each of the three criteria implemented in BMIRT had a better estimation of **α** and **δ** than the other methods with a low intertrait correlation (i.e., ρ = 0.2). However, when ρ = 0.5 and 0.8, MH with TRUE, AIC, or COR had larger RMSDs of **δ**. This was due to the inaccurate estimates of **δ**'s with some items. For example, MH with TRUE had the estimate of a δ of 1.1176 when the true parameter was −0.7087. The less accurate estimates of **δ** obtained by BMIRT led to the larger averaged RMSDs. Compared to the three BMIRT methods, HwG and the four IRTPRO procedures had an overall fairly accurate estimation of **α** and **δ** regardless of the intertrait correlations.

When *N* = 1000, *K* = 20, and ρ = 0.2, the three procedures implemented in BMIRT had a better estimate of **α** and **δ**. With an increase of the true intertrait correlation, HwG had an improved estimation of **α** and **δ** that is comparable to the three BMIRT methods. The four IRTPRO methods did not have a comparatively well estimation of **α**, but they had a fairly accurate estimation of **δ** if the true intertrait correlation was 0.2 or 0.8. Further, when *N* = 1000 and *K* = 40, BMIRT had larger RMSDs in estimating **δ**, indicating that it involved more error in estimating **δ** when the test length was longer, as described previously. HwG had an overall better estimation of **α** under the three intertrait correlation conditions. It performed well in estimating **δ** when ρ = 0.5. However, when ρ = 0.2 and 0.8, the four IRTPRO procedures outperformed HwG in estimating **δ**.

In terms of estimating ρ, the three procedures implemented in BMIRT performed similarly when the true intertrait correlation (ρ) was 0.2. However, when ρ increased, the AIC criterion had a worse estimation of ρ. This is due to the reason that the AIC criterion usually selected the model with a lower intertrait correlation coefficient (i.e., ρ = 0 or 0.1) to be the best model (i.e., the one with the smallest AIC value). Therefore, it had a worse estimation of ρ when the true intertrait correlation was moderate to strong. The TRUE criterion had an overall better estimation in all test situations. However, it is noted that in practice the actual intertrait correlation is usually unknown. Among the procedures where ρ is estimated, HwG had a relatively better performance in recovering ρ, especially when the true intertrait correlation was 0.5 or 0.8. When the dimensions or latent traits were not much correlated, HwG, COR, and the four IRTPRO procedures performed equally well.

In addition to RMSDs, biases were obtained and shown in the tables to determine the direction of estimation in each procedure. Specifically, a positive (negative) bias indicates that the estimate tends to be larger (smaller) than the true parameter. The results suggested that Gibbs, HwG, and the four procedures implemented in IRTPRO had in general an overestimate of **α**, while HwG tended to underestimate **δ**. However, the four procedures in IRTPRO had a positive bias in estimating **δ**_1_ (except for *N* = 1000, *K* = 20, ρ = 0.2) but a negative bias in estimating **δ**_2_. The three procedures in BMIRT tended to overestimate the model parameters when ρ was 0.2. When the intertrait correlation was larger, they could have negative biases for estimating **δ**. In terms of the bias of ρ, the four IRTPRO procedures and TRUE had in general positive biases (except for *N* = 500, *K* = 20, ρ = 0.5). AIC and COR tended to underestimate the intertrait correlation. HwG had positive biases when the true intertrait correlation was 0.2 or 0.8, but it had negative biases with the correlation being 0.5, regardless of sample sizes. Gibbs sampling had positive biases for all the parameters.

## 5. Discussion

In general, this study compared the performances in estimating multi-unidimensional GRMs using nine estimation methods. Simulation results indicate that the four methods implemented in IRTPRO (BAEM, ADQ, MHRM, BM) performed similarly in recovering **α**, **δ**, and ρ well under the simulated conditions. This result is consistent with those of Han and Paek (2014), where IRTPRO was used to estimate multi-unidimensional two-parameter models, of Wollack et al. (2002), where unidimensional nominal response models were evaluated via BAEM and BM procedures, and of Cai (2010a), where BAEM and MHRM methods were implemented to estimate the two-dimensional graded response model.

MH with the three criteria (TRUE, AIC, COR) executed using BMIRT also resulted in fairly accurate estimations of **α** and **δ**. However, these three criteria performed differently in estimating ρ based on the actual intertrait correlation. Specifically, these three criteria performed equally well when the latent traits had a weak correlation. However, in test situations where the intertrait correlation was moderate or high, only the TRUE criterion was able to obtain an accurate estimation of ρ. It is noted that the TRUE criterion is not applicable in many test situations due to the unknown true intertrait correlation, and hence is not practical with fitting such models. The COR criterion had a worse estimate than TRUE, but overall it had smaller RMSD's than the AIC criterion. Therefore, among the three criteria available from BMIRT, the COR criterion (i.e., using the correlation of subscales as the estimated intertrait correlation) is more applicable in real test situations and hence is preferred.

If the intertrait correlation is not of interest, HwG, MH with the three BMIRT criteria, and the four IRTPRO procedures can provide a fairly accurate estimation of **α** and **δ** with tests that are not very long (e.g., *K* < 40). This result agrees with the findings in Li et al. (2014) when they compared the TRUE criterion in BMIRT and the four procedures implemented in IRTPRO in estimating the multi-undimensional two-parameter model. However, if more items are adopted, MH with the three BMIRT criteria tends to result in a less accurate estimate of **δ** with some, if not all, items, and hence not suggested in situations with tests that have 40 or more items. Alternatively, one may consider HwG or any of the four IRTPRO procedures under this condition. If the dimensions are moderately correlated, the HwG algorithm has a relatively better estimate of **α** and **δ** than any of the four IRTPRO procedures. However, the four IRTPRO procedures may perform relatively better in estimating **δ** when the intertrait correlation is low or high. In general, if item discrimination parameters **α** or the intertrait correlation ρ are more of interest, one may consider implementing the HwG procedure.

In summary, the magnitude of the intertrait correlations may affect the performances of parameter recovery of these nine estimation methods. However, in practice, the actual values of these correlations are rarely known in advance. Hence, one may consider adopting the HwG procedure to estimate the intertrait correlations since it has an overall better estimation of ρ according to the simulation studies. If the correlations are moderate, HwG can perform better in estimating **α** and **δ** than the other procedures. If the estimated ρ is either high or low, the four procedures implemented in IRTPRO may be adopted if one is more interested in estimating **δ**. It is noted that, if the dimensions are lowly correlated, BMIRT can also be considered when the test length is not very long. However, if ρ is moderate or high, or if more items are adopted in a test, BMIRT is not recommended due to its inaccurate estimations of **δ** with some (if not all) items.

The simulation results in this study are based on test situations where two dimensions are involved. Further research can consider comparing these nine procedures in test situations with more than two dimensions. In addition, this study focuses on items with three-scale Likert scales and therefore two threshold parameters need to be estimated for each item. It is noted that the results may be limited to this specification and thus may not generalize to situations where five or seven categories are adopted. Further study can evaluate the estimation of these procedures using items with more than three scales or with different numbers of scales. Furthermore, Kuo and Sheng (2015) suggested that parameter estimate of the HwG procedure is not sensitive to different prior distributions for **α**. However, the selection of prior distributions for **α** may affect the estimation of the other estimation procedures. Therefore, further study can consider comparing these estimation methods using informative priors. Lastly, the simulation study only adopted 10 replications due to the computational expense of the MCMC procedures (i.e., Gibbs and HwG). Harwell et al. (1996) suggested that a minimum of 25 replications for Markov Chain studies in IRT-based research is recommended. Further studies can add more replications to achieve a better accuracy.

## Author contributions

TK conducted the simulation studies and summarized the conclusions. YS proofread and provided recommendations.

### Conflict of interest statement

The authors declare that the research was conducted in the absence of any commercial or financial relationships that could be construed as a potential conflict of interest.

## Footnotes

^1^Interested readers can contact the author for sample codes.

^2^Please see **Appendix A** for a sample code of implementing (1) in IRTPRO.

^3^Please see **Appendix B** for an example input file.

## Appendix A

Sample code for fitting the multi-unidimensional GRM using IRTPRO using the BAEN estimation, where *N* = 1000, *K* = 20 and ρ = 0.2:

  Project:
      Name = Y_IRTPRO11;
  Data:
      File = .\Y_IRTPRO11.ssig;
  Analysis:
      Name = Test1;
      Mode = Calibration;
  Title:
      Simulation study;
  Comments:
      GRM with two subtests, with 10 items
                          in each;
  Estimation:
      Method = BAEM;
      E-Step = 500, 1e-005;
      SE = S-EM;
      M-Step = 50, 1e-006;
      Quadrature = 49, 6;
      SEM = 0.001;
      SS = 1e-005;
  Scoring:
      Mean = 0;
      SD = 1;
  Miscellaneous:
      Decimal = 2;
      Processors = 4;
      Print CTLD, P-Nums, Diagnostic;
      Min Exp = 1;
  Groups:
  Group :
      Dimension = 2;
      Items = VAR1, VAR2, VAR3, VAR4, VAR5,
      VAR6, VAR7, VAR8, VAR9, VAR10,
      VAR11, VAR12, VAR13, VAR14, VAR15,
      VAR16, VAR17, VAR18, VAR19, VAR20;
      Codes(VAR1) = 0(0), 1(1), 2(2);
      Codes(VAR2) = 0(0), 1(1), 2(2);
      Codes(VAR3) = 0(0), 1(1), 2(2);
      Codes(VAR4) = 0(0), 1(1), 2(2);
      Codes(VAR5) = 0(0), 1(1), 2(2);
      Codes(VAR6) = 0(0), 1(1), 2(2);
      Codes(VAR7) = 0(0), 1(1), 2(2);
      Codes(VAR8) = 0(0), 1(1), 2(2);
      Codes(VAR9) = 0(0), 1(1), 2(2);
      Codes(VAR10) = 0(0), 1(1), 2(2);
      Codes(VAR11) = 0(0), 1(1), 2(2);
      Codes(VAR12) = 0(0), 1(1), 2(2);
      Codes(VAR13) = 0(0), 1(1), 2(2);
      Codes(VAR14) = 0(0), 1(1), 2(2);
      Codes(VAR15) = 0(0), 1(1), 2(2);
      Codes(VAR16) = 0(0), 1(1), 2(2);
      Codes(VAR17) = 0(0), 1(1), 2(2);
      Codes(VAR18) = 0(0), 1(1), 2(2);
      Codes(VAR19) = 0(0), 1(1), 2(2);
      Codes(VAR20) = 0(0), 1(1), 2(2);
      Model(VAR1) = Graded;
      Model(VAR2) = Graded;
      Model(VAR3) = Graded;
      Model(VAR4) = Graded;
      Model(VAR5) = Graded;
      Model(VAR6) = Graded;
      Model(VAR7) = Graded;
      Model(VAR8) = Graded;
      Model(VAR9) = Graded;
      Model(VAR10) = Graded;
      Model(VAR11) = Graded;
      Model(VAR12) = Graded;
      Model(VAR13) = Graded;
      Model(VAR14) = Graded;
      Model(VAR15) = Graded;
      Model(VAR16) = Graded;
      Model(VAR17) = Graded;
      Model(VAR18) = Graded;
      Model(VAR19) = Graded;
      Model(VAR20) = Graded;
      Means = 0.0, 0.0;
      Covariances = 1.0,
                    Free, 1.0;
  Constraints:
      (VAR1, Slope[1]) = 0.0;
      (VAR2, Slope[1]) = 0.0;
      (VAR3, Slope[1]) = 0.0;
      (VAR4, Slope[1]) = 0.0;
      (VAR5, Slope[1]) = 0.0;
      (VAR6, Slope[1]) = 0.0;
      (VAR7, Slope[1]) = 0.0;
      (VAR8, Slope[1]) = 0.0;
      (VAR9, Slope[1]) = 0.0;
      (VAR10, Slope[1]) = 0.0;
      (VAR11, Slope[0]) = 0.0;
      (VAR12, Slope[0]) = 0.0;
      (VAR13, Slope[0]) = 0.0;
      (VAR14, Slope[0]) = 0.0;
      (VAR15, Slope[0]) = 0.0;
      (VAR16, Slope[0]) = 0.0;
      (VAR17, Slope[0]) = 0.0;
      (VAR18, Slope[0]) = 0.0;
      (VAR19, Slope[0]) = 0.0;
      (VAR20, Slope[0]) = 0.0;
 

## Appendix B

Example batch file for implementing the MH estimation via BMIRT using the TRUE method, where *N* = 1000, *K* = 20, ρ = 0.2:

     1000 20 1 1.0 1.0 10000 5000 2 5 9238796
     31 0.0 1.0 0.3 0.5 0.15
     1.5 1.5 0.01 0.0 1.5 0.01 100 400 0.01
     0.3 0.05 1.0 0.2 0.05 0.05
     1.0 0.3
     
     33333333333333333333
     1 2
     1  2  3  4  5  6  7  8  9 10 11 12 13 14
     15 16 17 18 19 20
     11111111110000000000
     00000000001111111111
     1 0.2 1 0 0
 

Please refer to Yao (2003) for detailed information regarding the input arguments.
